# Taylor County Medical Society

**Published:** 1890-05

**Authors:** Hicks Florence

**Affiliations:** Secretary


### Society Notes.

For Daniel's Texas Medical Journal.

### TAYLOR COUNTY MEDICAL SOCIETY.

The Taylor County Medical Society met at 11 o'clock, a. m., at Dr. Grizzard's office as per appointment.

Physicians present: Drs. Rodman, Richardson, Blakemore, Isbell, Bryce, Rogers, Harrington and Florence.

First in order of business, was the report of committees. The committee appointed to wait upon Dr. Robert E. Lemond, an advertising specialist, also, an honorary member of the society, to inform him of his unprofessional manner of practicing, reported a formal resignation sent in by the said Dr. Robert F. Lemond, which the society declined to accept, and his name was dropped from the Society.

Dr. Rodman read an interesting essay—subject, La Grippe, which was ably and thoroughly discussed. The discussion terminated in personal experiences of the members with anti-pyrin and anti-febrin.

The Society were divided in their opinions respecting the safer aud more useful drug of the two.

Dr. Harrington reported a case of placental polypus. He stated that placenta remained in utero for some months after an abortion, and had preserved its vitality perfectly.

At the next meeting Dr. Boyce will read a paper on dysentery.

The Society adjourned to meet on the first Saturday in June at Dr. Harrington's office.

We are agitating the subject of organizing a District Medical Association in the near future.

HICKS FLORENCE, M. D., Secretary.

---

JIMMIE WAS ALL RIGHT.—"In my neighborhood," said the doctor, dropping into a chair and lazily throwing one leg over the other, "there was a nasty little cock-eyed bricklayer named Lynch. He was a "Hinglishman," he said, "from 'Arrowgate." His wife was a pretty decent sort of fellow; but he was too mean to eat enough. He had a way of coming over to the drug store and asking Bob what was good for so and so. He never sent for me in his life, never bought over ten cents worth of anything in the drug store. His "big holt" was "Seen-na and Salts." Jimmie his son was down with chill and fever and he was giving him calomel, and about three grains of quinine a day; but Jimmie got no better,—fast. About the fourth chill Jimmie had, they gave in, and sent for me. I prescribed *enough* quinine, and prevented the paroxysm. At my next visit I found him well, and I accordingly said: 'Jimmie is all right now; he may get up to-morrow.'

"Yes, *Jimmie's* all right," said his mother, "I knowed that last dose of calamy I gi' him would set *Jimmie* all right."

"I went out and kicked myself," said the doctor.

"Lynch had a dog, and would'nt feed him. The dog, thrown on his own resources, used to go hunting for young rabbits, which in summer were plentiful even on the out-skirts of the town. Lynch saw him with a rabbit one day and *took it away from him*, —fact. Talk about mean men;" and the doctor looked too disgusted for anything.

---

DRS. W. J. LANE AND ASA W. POPE of Marshall, are at the New York Polyclinic.

